# Clinical variant interpretation and biologically relevant reference transcripts

**DOI:** 10.1038/s41525-022-00329-6

**Published:** 2022-10-18

**Authors:** Fernando Pozo, José Manuel Rodriguez, Jesús Vázquez, Michael L. Tress

**Affiliations:** 1 Bioinformatics Institute, Spanish National Cancer Research Centre (CNIO), 28029 Madrid, Spain; 2 Cardiovascular Proteomics Laboratory, Centro Nacional de Investigaciones Cardiovasculares Carlos III (CNIC), 28029 Madrid, Spain; 3 CIBER de Enfermedades Cardiovasculares (CIBERCV), 28029 Madrid, Spain

**Keywords:** Genome informatics, Clinical genetics, Genome evolution, Clinical genetics

## Abstract

Clinical variant interpretation is highly dependent on the choice of reference transcript. Although the longest transcript has traditionally been chosen as the reference, APPRIS principal and MANE Select transcripts, biologically supported reference sequences, are now available. In this study, we show that MANE Select and APPRIS principal transcripts are the best reference transcripts for clinical variation. APPRIS principal and MANE Select transcripts capture almost all ClinVar pathogenic variants, and they are particularly powerful over the 94% of coding genes in which they agree. We find that a vanishingly small number of ClinVar pathogenic variants affect alternative protein products. Alternative isoforms that are likely to be clinically relevant can be predicted using TRIFID scores, the highest scoring alternative transcripts are almost 700 times more likely to house pathogenic variants. We believe that APPRIS, MANE and TRIFID are essential tools for clinical variant interpretation.

## Introduction

Determining which transcripts are most likely to produce functionally important proteins is not a simple task. Protein coding genes can have large numbers of alternatively spliced transcripts, so clinical researchers are often faced with a complex choice of coding transcripts when they detect a new variant. The American College of Medical Genetics (ACMG) Laboratory Quality Assurance Committee recommends mapping new variants to the “most common human transcript, largest known transcript, or tissue-specific alternatively spliced transcript”^1^, and referring this transcript to a reference transcript, which could be either the longest transcript or the most clinically relevant transcript^2^ in RefSeq^3^. They warn against over-interpreting truncating variants in alternative exons^2^ and urge caution when assigning clinical significance to variants in exons where no other pathogenic variants exist. However, they also suggest laboratories do not limit themselves to the longest transcript^2^.

Although the longest transcript is often used to represent coding genes, it does not always produce the most biologically relevant isoform. The gene *TAFAZZIN* is a good example. The gene product, tafazzin, named after an Italian comic^4^, is an acyltransferase that regulates cardiolipin in the mitochondrial membrane^5^, Variants cause Barth Syndrome^4^, a disorder that can lead to cardiomyopathy and skeletal muscle weakness^6^.

The UniProtKB database^7^ usually selects the longest isoform to represent coding genes. From 1997 until 2022, the UniProtKB representative for *TAFAZZIN* (previously, *TAZ*) was a protein of 292 amino acid residues known as the “full-length” isoform. In 2003, Vaz et al.^8^ produced an analysis succinctly titled “Only one splice variant of the human TAZ gene encodes a functional protein with a role in cardiolipin metabolism”, and it was not the “full length” isoform. They found that the functional tafazzin protein was generated from a transcript that skipped exon 5. Further research^5,9^ supported this.

**Fig. 1:**
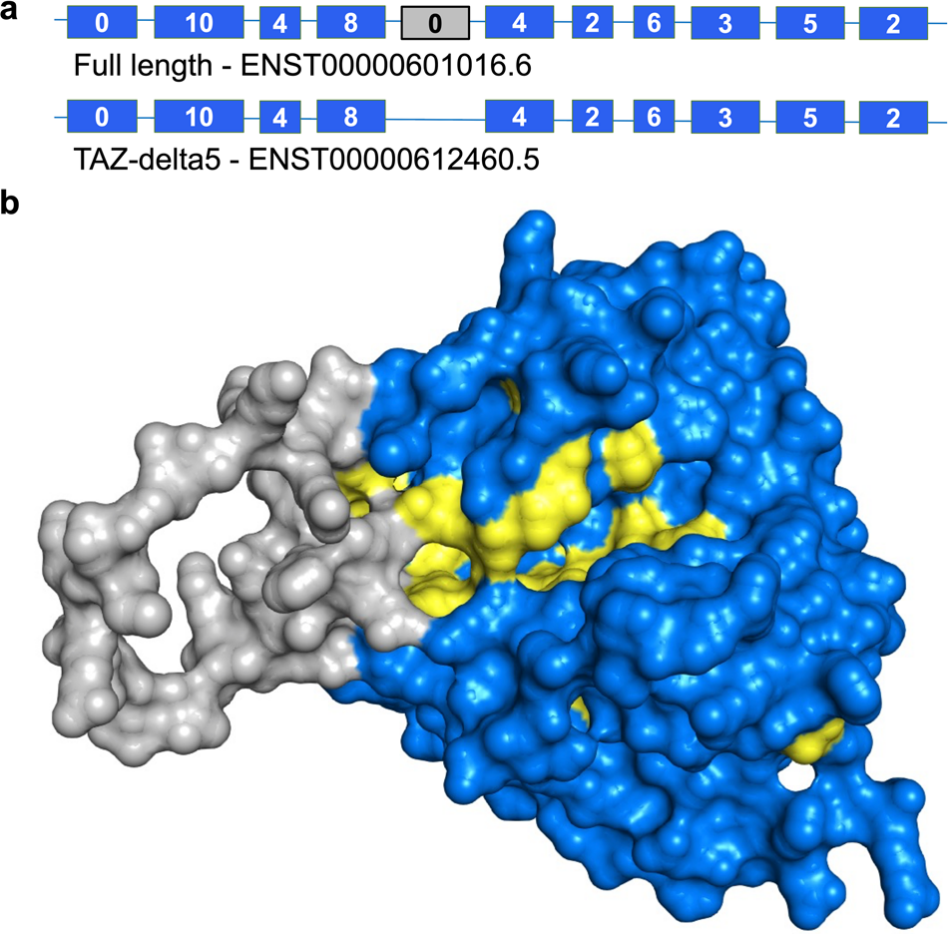
The “full-length” splice variant of *TAFAZZIN*. **a** The two most studied *TAFAZZIN* transcripts. The “full-length” transcript has 11 exons, the likely main functional transcript (TAZ-delta5) has ten. The inserted exon 5 is shown with a grey background. Exons are not to scale. The number of ClinVar^10^ pathogenic and likely pathogenic variants that map to each exon is shown inside the exon. Only exon 1 (the trans-membrane helix) and exon 5 have no ClinVar pathogenic variants. **b** The model of the “full-length” isoform of *TAFAZZIN* generated by AlphaFold^45^ for UniProtKB, showing the predicted protein surface. The inserted loop from exon 5 is shown in grey. Residues coloured in yellow are 100% conserved in an alignment of tafazzin from 10 species, human, mouse, zebrafish, fruit fly, cockroach, orbweaver spider, chickpea, potato, mushroom and slime mould. These are the most important residues in the binding cleft/catalytic site region. The inserted loop blocks this region.

In fact, exon 5 seems highly unlikely to be functionally important. The 5′ end of the exon derives from a SINE Alu transposon and is conserved only in apes. ClinVar^10^, the main repository of clinically significant human variants and their phenotypes, annotates 44 pathogenic variants in Barth Syndrome. None map to exon 5 of the full-length transcript (Fig. 1). The region translated from exon 5 is likely to be unstructured and to disrupt the globular structure of the protein (the grey loop in Fig. 1). It would also interfere with a region conserved across plants, fungi and animals (the yellow residues in Fig. 1).

The choice of the full-length transcript as reference has had difficult-to-remediate downstream effects in other databases and large-scale analyses. For a start, the equivalent of the human exon is annotated in mammalian species as far away as beaver, whale, and elephant, even though the exon itself is primate-derived. In addition, the Pfam database^11^ incorporates the full-length isoform of *TAFAZZIN* into its acyltransferase domain definition, and the Locus Reference Genomic^12^ chooses the full-length transcript as the most clinically important, in part because many (largely benign) variants have been mapped to exon 5. The accumulated downstream evidence for the *TAFAZZIN* “full length” transcript is such that Ensembl^13^ and RefSeq annotators have chosen it as the MANE Select transcript^14^ for this gene. The presence of the full-length splice variant of *TAFAZZIN* in these (and other) databases cements its reputation as the most important splice variant. However, this is classic circular evidence, and difficult to undo.

Here we analyse the effectiveness of two methods recently developed to select reference splice variants, APPRIS^15^ and MANE^14^. MANE Select reference transcripts, developed by Ensembl and RefSeq annotators, are based on evidence such as expression level, evolutionary conservation, and clinical variants. The annotation pipeline also considers the UniProtKB display and APPRIS principal isoform. We developed the APPRIS database. It chooses principal isoforms based on cross-species conservation and the preservation of protein features^16^.

## Results

### Mapping ClinVar variants to coding exon sets

We mapped ClinVar variants to the coding exons (CDS) of three sets of reference transcripts, APPRIS “principal transcripts”, MANE Select transcripts and the longest CDS of each coding genes (see methods for details). The fine details of the mapping and analysis are detailed solely for the APPRIS principal transcripts, but we carried out the same process of computer analysis and manual curation for all three sets.

### Only a handful of pathogenic variants map to alternative exons

**Fig. 2:**
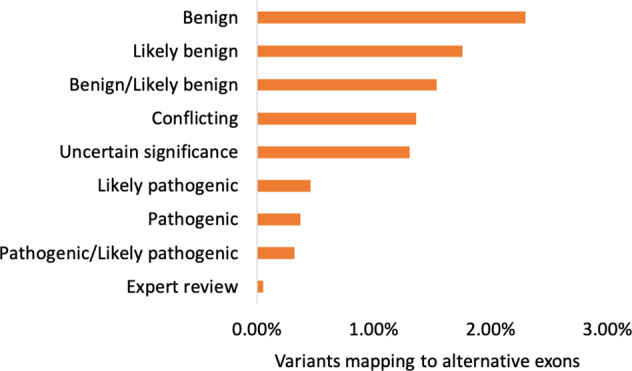
The percentage of ClinVar variants that map exclusively to alternative exons. The small percentage of variants that do not map to APPRIS principal exons. Variants are grouped by ClinVar labels; the labels correspond to the CLIN_SIG entry. “Expert review” are variants labelled as “reviewed by expert panel”. Alternative exons make up 2.43% of all coding nucleotides, but <0.5% of all variants labelled with the word “pathogenic” fall in alternative exons.

Just 2.43% of GENCODE v37^17^ coding nucleotides are wholly alternative (do not overlap APPRIS principal transcripts), and considerably fewer ClinVar variants map to these nucleotides than would be expected by chance (1.6%). Variants can be distinguished by clinical significance (Fig. 2). We found the more damaging the ClinVar clinical significance label, the fewer variants mapped to alternative exons. For example, while 2.3% of variants tagged as “Benign” mapped to alternative exons, the same was true of just 1.31% of “Uncertain Significance” variants. Very few “Pathogenic” variants mapped to alternative exons (0.37%). Pathogenic variants that have undergone expert curation are even less likely to map to alternative exons than ordinary pathogenic variants. Just five of 9,491 variants reviewed by an expert panel (0.05%) mapped to alternative nucleotides.

Restricting variants to those supported by PubMed references magnifies the differences. The proportion of pathogenic and likely pathogenic variants mapping to alternative exons decreases for variants with PubMed references (from 0.37% to 0.22% for variants tagged as “Pathogenic”, and from 0.46% to 0.25% for variants tagged as “Likely pathogenic”), while the proportion of “Benign” variants in alternative exons increases (2.29% to 2.94%).

### Manual curation of pathogenic variants in APPRIS alternative exons

**Fig. 3:**
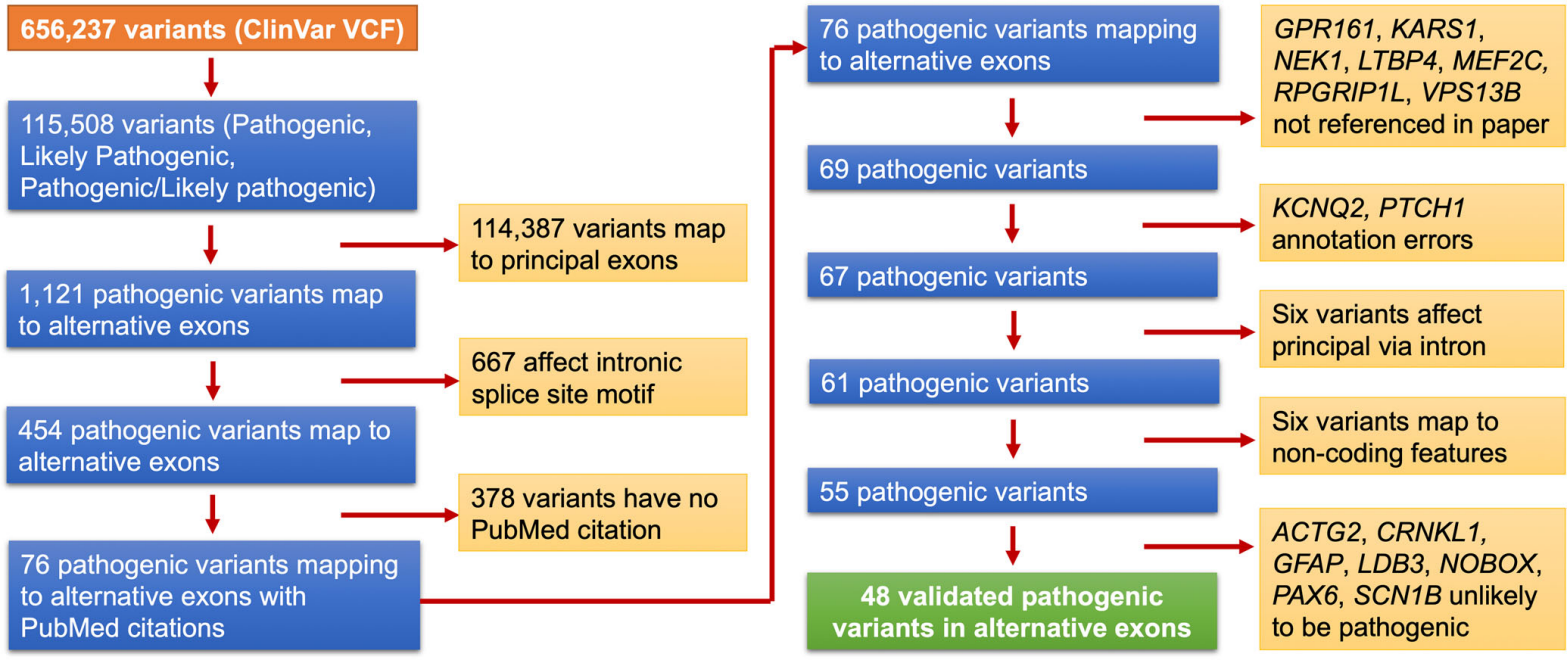
The process of validating the pathogenic variants that map to alternative exons. The process of mapping pathogenic variants from the ClinVar VCF file (version 4th April 2021) to the APPRIS principal and alternative transcripts (left side of the flow chart), and the breakdown of the manual analysis of the 76 pathogenic variants that map uniquely to APPRIS alternative variants (right side of the chart). We found that just 48 pathogenic variants had a direct effect on the expressed alternative protein. We tagged these pathogenic variants as “validated”.

We mapped ClinVar pathogenic variants with PubMed support to exons and splice sites from APPRIS principal transcripts (see methods). We defined “pathogenic variants” as those variants tagged as Pathogenic, Likely pathogenic or Pathogenic/Likely pathogenic in ClinVar. There were 115,508 pathogenic variants, 17.6% of all variants annotated in ClinVar (Fig. 3). Coding exons from APPRIS principal transcripts captured 114,387 of these variants (99.03%).

Most of the 1211 pathogenic variants that map to alternative coding exons are found in 5′ or 3′ exon extensions within just a few bases of principal coding exon splice sites. Although they map to alternative coding exons, these variants are much more likely to affect the splice site of the principal transcript. To account for this, we carried out an intronic splice site motif aware mapping of ClinVar variants by extending coding exons in APPRIS principal transcripts by three nucleotides at the 5′ end, and by 5 nucleotides at the 3′ end. The 667 pathogenic variants that mapped to these extended principal CDS counted as mapping to reference transcripts rather than to alternative exons, meaning that the total of pathogenic variants captured by APPRIS principal transcripts rose to 115,054 (99.61%). Alternative exons captured just 454 pathogenic variants and 76 of these had PubMed support.

The ClinVar database does not assign pathogenicity labels. Instead, these are determined by the submitting group following guidelines issued by the ACMG. The submission guidelines have changed over time. Given the changing guidelines and the possibility of human error, some pathogenic variants may be erroneous. We carried out a manual curation of these 76 variants, reviewing the supporting publications to determine whether the variant was correctly transferred between research paper and clinical database, and whether it affected the translated protein.

We removed seven pathogenic variants from the list because they were not mentioned in the PubMed papers to which they were linked (Fig. 3). In addition, two of the variants appear to be annotation errors. The coordinates of the pathogenic variant in *KCNQ2*^18^ seem to have been erroneously mapped to an alternative exon, and in *PTCH1* the authors cannot have not carried out confirmatory experiments using the equivalent to the alternative exon in fish^19^ because this exon is not conserved outside of primates.

We eliminated six pathogenic variants because the supporting PubMed papers found that the effect of the variant was actually on the splicing or the expression of the main transcript^20,21^ rather than the alternative transcript, while a further six pathogenic variants affected non-coding features, including three that mapped to a nonsense-mediated decay (NMD) exon in *SNRPB*^22^. The variant in *HBD* mapped to a GATA1 binding site^23^, and the variant in *SDCCAG8* affected an exonic splicing enhancer^24^. The alternative coding exons the variants mapped to in all these cases are only conserved in primates and have little transcript support.

**Fig. 4:**
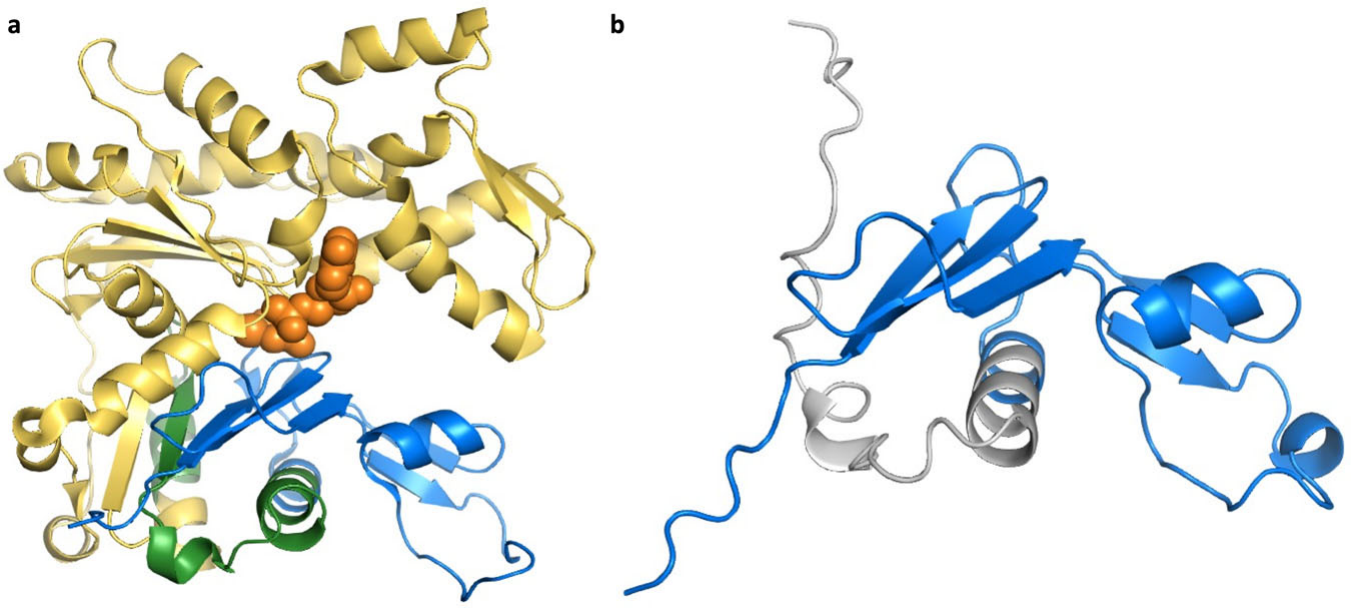
The effect of *ACTG2* alternative splice event on protein structure. **a** The sequence of alternative isoform from the novel exon mapped onto the structure of chicken smooth muscle actin (PDB: 3W3D, 28). The region that is maintained in the alternative isoform is shown in blue, while the 34 residues that would be replaced by the novel primate exon with the pathogenic variant are shown in green. The remainder of the structure (in yellow) would be lost from this presumed protein. The ATP and calcium bound by the chicken actin protein (lost in the alternative isoform) are in orange space fill. Mapping was carried out using HHPRED^47^. **b** A model of the same truncated *ACTG2* isoform generated by AlphaFold^45^. The maintained sequence is in blue, the novel predicted region is in grey. Despite the AlphaFold prediction, the substituted sequence is unlikely to fold into an extended helix, and will certainly not bind ATP. Both images were generated with PyMol.

Finally, we believe the authors of seven research papers have mistakenly classified the variant as having a pathogenic effect via the alternative protein. One example is in the gene *ACTG2*, which produces smooth muscle actin, a protein that is even 95% identical between vertebrates and invertebrates. The predicted pathogenic variant^25^ affects a novel 3′ exon derived from a LINE2 transposon that is conserved only in chimpanzee. The isoform produced from this novel exon would be missing almost three-quarters of the actin fold (Fig. 4). It is hard to imagine how a variant in an exon that produces a truncated protein isoform could affect megacystis-microcolon-intestinal hypoperistalsis syndrome, a severe disorder that affects bladder and intestine muscles^25^. Especially since the transcript appears not to be expressed in any tissue^26^, and certainly not in bladder or intestines. The pathogenicity of this variant is solely supported by association studies, and the authors even admit that “the data from this family suggests but perhaps do not prove entirely that the alternative exon 4… is functionally important”.

The variant in the primate-derived alternative exon of *LDB3* would change an isoleucine for a methionine residue. Expression of the exon is limited to testis, and there is no evidence it is expressed in heart^26^. This is incongruent, given that the variant is supposed to cause dilated cardiomyopathy^27^. *LDB3* reference sequences in both UniProtKB and the Locus Reference Genomic incorporate this exon, presumably because of this unlikely pathogenic variant.

**Fig. 5:**
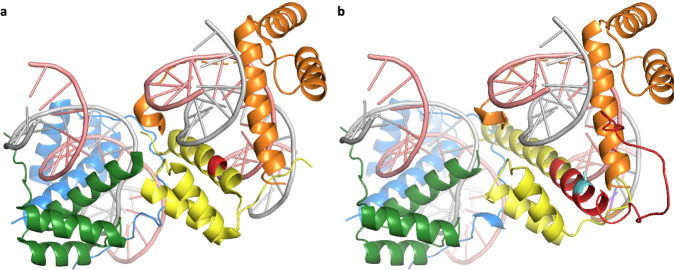
The effect of the *NOBOX* 3′ splice site extension on protein structure. **a** The image shows the crystal structure of *Drosophila melanogaster Aristaless* and *Clawless* homeodomain proteins bound to DNA (PDB: 3A01). The *Aristaless* protein (chain F, yellow) has 57% identity to the *NOBOX* homeobox domain. The residues where the 32 amino acid insert would break the *NOBOX* homeobox domain are highlighted in red. The insertion is right next to the conserved DNA-binding residues of the homeobox domain and this primate-derived exon would almost certainly banish the *NOBOX* homeobox DNA-binding function. **b** The same PDB structure with the inserted exon modelled by AlphaFold for the UniProtKB *NOBOX* display isoform grafted onto the structure. The inserted exon is in red. The predicted effect of the insertion would be to extend the helix (again) and to interfere with the DNA-binding of the human *Aristaless* homologue. Mapping was carried out using HHPRED and both images were generated with PyMol.

The variant in the *NOBOX* alternative transcript, ENST00000467773.1, was assumed by the authors to be pathogenic^28^ because it falls within a conserved homeobox domain. However, the variant, a conservative serine to threonine swap, maps to a little expressed^26^, primate-derived alternative exon that itself inserts into the region that produces the homeobox domain (Fig. 5). The inserted exon would almost certainly disrupt the domain, particularly since the inserted exon is adjacent to the conserved asparagine and arginine residues that bind DNA (Fig. 5). This novel exon almost certainly would eliminate DNA binding with or without the variant. ENST00000467773.1 is the longest CDS. It is also the MANE Select transcript and produces the UniProtKB display isoform, in part because of this erroneous variant.

At the end of our analysis, we found that PubMed publications validated a pathogenic effect on the alternative protein product for 48 of the annotated pathogenic variants (see Supplementary Table 1). The 48 variants mapped to 30 different alternative transcripts. These 48 “validated” variants are just 0.138% of all 34,833 PubMed-supported pathogenic variants.

Almost all of the 28 PubMed-supported pathogenic variants that mapped to alternative exons but that did not affect the alternative protein were primate-derived, many only across higher primates. Recently evolved alternative exons are highly unlikely to have gained sufficient functional importance for variants to have a pathogenic effect on the protein product^29^, and in order to be considered pathogenic should have enhanced supporting evidence. For example, although the alternative exon that houses the *REEP6* pathogenic variant evolved recently, during the eutherian clade, its pathogenicity is also supported by expression data. The variant is predicted to cause autosomal-recessive retinitis pigmentosa^30^, and the alternative isoform is the main isoform in retina^31^.

### Pathogenic variants in exons alternative to MANE Select and longest CDS transcripts

**Fig. 6:**
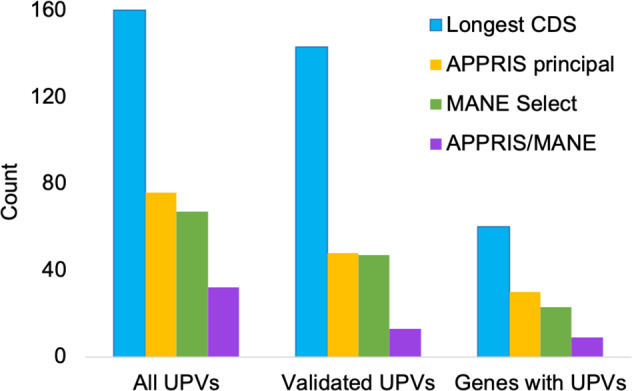
Pathogenic variants not captured by reference transcripts. ClinVar Pathogenic, Likely pathogenic and Pathogenic/Likely pathogenic variants with PubMed support that are not captured by reference transcripts are here termed uncaptured pathogenic variants (UPVs). The method for reference transcript selection is shown in the legend. “All UPVs” are all pathogenic variants with PubMed citations that are not captured by the reference transcripts, “Validated UPVs” are those uncaptured pathogenic variants with PubMed support that were validated by manual curation, “Genes with UPVs” are the number of distinct genes with validated uncaptured pathogenic variants.

We also analysed the relationship between pathogenic variants and the two other methods for selecting reference sequences, MANE Select transcripts and the longest CDS (see Supplementary Tables 2, 3). We mapped the 115,508 pathogenic variants from the ClinVar VCF file to both these sets of reference transcripts as we had with the principal transcripts (Fig. 3). Prior to manual curation, all but 67 of the 33,736 pathogenic variants with PubMed support mapped to MANE Select transcripts rather than alternative transcripts (Fig. 6). This was similar to APPRIS, and indeed most of the pathogenic variants in alternative exons coincided. The longest CDS captured all but 160 of the pathogenic variants supported by PubMed publications (Fig. 6).

As we had with the pathogenic variants that mapped to APPRIS alternative exons, we validated the likely pathogenic effect on the alternative protein products for variants that were in exons alternative to MANE Select transcripts, and those that were in exons alternative to the longest CDS. We validated 47 of the 67 pathogenic variants that mapped to MANE Select alternative exons (Fig. 6). This was 0.139% of PubMed-supported pathogenic variants, similar to the 0.138% of validated pathogenic variants that mapped to APPRIS alternative transcripts. Unsurprisingly, there was no significant difference between the proportion of pathogenic variants captured by APPRIS principal transcripts and those captured by MANE Select transcripts,

The longest CDS was less successful at capturing validated pathogenic variants. After curation, 143 pathogenic variants from 60 genes (Fig. 6) mapped to transcripts with shorter CDS (0.411% of pathogenic variants with PubMed support). The longest CDS miss three times as many validated pathogenic variants as the APPRIS principal and MANE Select transcripts, even though they cover substantially more nucleotides. This difference is clearly significant (two-tailed Fisher exact test, *p* < 0.00001). In fact, over the whole of ClinVar, the longest CDS fails to pick up 535 pathogenic variants.

APPRIS principal and MANE Select agree on the reference transcript over a total of 2,985 genes that have PubMed-supported pathogenic variants. Within these genes, the MANE and APPRIS supported reference transcripts captured 31,259 of 31,291 PubMed-supported pathogenic variants (99.9%). Just 32 PubMed-supported pathogenic variants mapped to alternative exons (Supplementary Table 4), and of these, we validated just 13 (0.042%) via their PubMed references. When they agree, APPRIS principal and MANE Select transcripts capture almost all annotated clinically important variants.

The same is not true for the longest CDS. Over the set of genes where MANE Select and APPRIS principal transcript coincide, the longest CDS fails to capture 113 validated pathogenic variants. Ten of these are also missed by APPRIS/MANE. That means that over those genes where the longest CDS does not agree with the APPRIS principal and MANE Select reference, the longest CDS fails to capture 103 pathogenic variants, and the MANE Select/APPRIS principal reference just three (in *REEP6*, *SLC25A3* and *TCF3*). Even counting the pathogenic variant in *TCF3* (where the longest CDS is almost certainly not biologically relevant), this is still a ratio of 34 to 1.

### Extending the analysis to all pathogenic variants

To quantify the alternative transcripts with ClinVar pathogenic variants, we extended our analysis to include all pathogenic variants, regardless of whether they were supported by a publication. To guarantee that pathogenic variants mapped to alternative transcripts, we did not use MANE or APPRIS to select reference transcripts, and instead tried to map as many pathogenic variants as possible to a single transcript with each gene. Pathogenic variants that were not captured by this transcript were deemed to map to alternative exons.

In this analysis of all ClinVar pathogenic variants, these variants mapped to alternative exons in just 67 transcripts. Because many of these pathogenic variants were without PubMed support, we “validated” the likely effect on the protein by determining the relative age and expression levels of the alternative exons that held the variants. As we have already shown, pathogenic variants in recently evolved exons with little or no transcript support are not likely to have an effect on protein products.

Pathogenic variants mapped to primate-derived exons in 34 alternative transcripts. These 34 primate-derived exons had little or no transcript support^26^. For 10 of the variants, we have already shown that their PubMed references do not support an effect on the alternative protein (*ACTG2*, *HBD*, etc.). Ten of the 34 derived from primate transposons, seven were NMD targets, including all three alternative exons in *SCN1A*, and one is no longer annotated as coding. We eliminated these alternative transcripts as “not validated”.

**Fig. 7:**
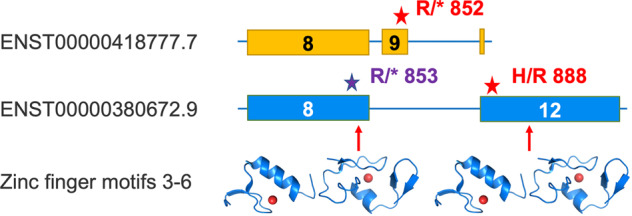
Mis-annotation of a pathogenic variant in *BNC2*. The figure represents the 3′ coding exons of two *BNC2* transcripts (not to scale). Exon numbers (shown inside each exon) are from their position in the GENCODE v37 reference set. The position of annotated and experimental pathogenic variants (stars) is marked next to the corresponding transcripts. Exons 8 and 12 in ENST00000380672.9 (the APPRIS principal and MANE Select transcript) code for four zinc finger motifs. Inserted exon 9 in transcript ENST00000418777.5 leads to a frame change and a premature stop codon, which would eliminate three of the motifs. Below the two exons, the motifs are represented by the PDB structures of 3MJH and 1WJ0, mapped using HHPRED. *BNC2* has two pathogenic variants in ClinVar (red stars). One is annotated in ENST00000380672.9 and produces a histidine for arginine swap at amino acid residue 888. The other is annotated exon 9 of transcript ENST00000418777.5, and is predicted to affect residue 852 of the alternative isoform. The experimentally determined pathogenic variant (purple star), which would affect both transcripts, is reported to change an arginine to a stop codon at residue 853 of the principal isoform.

Twelve of the 33 remaining alternative transcripts with pathogenic variants had PubMed support. However, a literature search for the variant in the alternative transcript in *BNC2* turned up an annotation error. *BNC2* has two annotated pathogenic variants (Fig. 7). One variant is in the final coding exon of ENST00000380672.9, the APPRIS principal and MANE Select transcript. This variant produces a histidine for arginine swap at amino acid residue 888 and would banish the zinc binding of the 2nd of four C-terminal zinc-binding motifs. The other pathogenic variant is in an inserted exon in transcript ENST00000418777.5. The inserted exon leads to a frame change and a premature stop codon. The variant is predicted to produce a premature stop codon at residue 852 of the alternative isoform (Fig. 7); a premature stop codon in an already truncated transcript. Both the truncations would eliminate three of the four C-terminal zinc binding motifs (Fig. 7).

Although this variant is not supported by any publication, there is a third experimentally determined pathogenic variant for *BNC2* not annotated in ClinVar. It too produces a premature stop codon, but at arginine 853 of the principal isoform, not arginine 852 of the alternative isoform^32^. This stop codon would also almost certainly be pathogenic since it would remove all four C-terminal zinc binding motifs. It seems that this pathogenic variant was mapped to ENST00000418777.5 erroneously during the lift-over from GRCh37 to GRCh38^33^. We also removed *BNC2* from the set of genes with pathogenic variants in more than one distinct transcript.

The genes with validated pathogenic variants in alternative transcripts are detailed in Supplementary Table 5, while those genes with pathogenic variants that we did not validate are listed in Supplementary Table 6. There are 32 alternative transcripts in total, and three genes, *GCNT2, GNAS* and *PCDH15*, have pathogenic variants in three distinct transcripts (Table 1). So, validated pathogenic variants map to more than one transcript in just 29 of the 11,132 genes with pathogenic variants in ClinVar.

**Table 1:** List of human alternative transcripts that harbour validated pathogenic variants.

Gene name	Gene ID	Alternative transcript ID	TRIFID	PUBMED support	MPC	Protein effect
*ASPH*	*ENSG00000198363.18*	*ENST00000389204.8*	0.426	−	−	C-terminal swap
*CACNA1C*	*ENSG00000151067.22*	*ENST00000399641.6*	0.979	Yes	Yes	Homologous substitution
*CACNA1D*	*ENSG00000157388.19*	*ENST00000422281.7*	0.885	−	Yes	Homologous substitution
*CDKN2A*	*ENSG00000147889.18*	*ENST00000579755.2*	1	−	Yes	Two different proteins
*COL6A2*	*ENSG00000142173.16*	*ENST00000397763.5*	0.587	−	−	C-terminal swap
*DST*	*ENSG00000151914.21*	*ENST00000370765.11*	0.405	Yes	Yes	C-terminal swap
*ERCC6*	*ENSG00000225830.16*	*ENST00000515869.1*	0.823	Yes	Yes	C-terminal swap
*FGFR2*	*ENSG00000066468.23*	*ENST00000457416.6*	0.717	Yes	−	Homologous substitution
*GCNT2*	*ENSG00000111846.19*	*ENST00000265012.5*	0.736	−	Yes	Homologous substitution
*GCNT2*	*ENSG00000111846.19*	*ENST00000640968.1*	0.691	−	Yes	Homologous substitution
*GNAS*	*ENSG00000087460.28*	*ENST00000453292.6*	0.229	−	Yes	Two different proteins
*GNAS*	*ENSG00000087460.28*	*ENST00000676826.1*	0.153	−	−	Homologous substitution
*GRIA3*	*ENSG00000125675.19*	*ENST00000541091.5*	0.943	–	Yes	Homologous substitution
*IQSEC2*	*ENSG00000124313.18*	*ENST00000375365.2*	0.7	−	Yes	N-terminal swap
*ITGA6*	*ENSG00000091409.15*	*ENST00000458358.5*	0.284	Yes	−	Homologous substitution
*KRAS*	*ENSG00000133703.13*	*ENST00000256078.10*	1	−	−	Homologous substitution
*LAMA3*	*ENSG00000053747.17*	*ENST00000587184.5*	0.217	Yes	Yes	N-terminal swap
*MASP1*	*ENSG00000127241.18*	*ENST00000296280.11*	0.713	Yes	Yes	Homologous substitution
*MECP2*	*ENSG00000169057.24*	*ENST00000303391.11*	0.996	−	Yes	N-terminal swap
*MOCS2*	*ENSG00000164172.20*	*ENST00000584946.5*	0.417	Yes	Yes	Two different proteins
*NEB*	*ENSG00000183091.20*	*ENST00000427231.7*	0.657	−	Yes	Homologous substitution
*OTOF*	*ENSG00000115155.19*	*ENST00000403946.7*	0.942	Yes	Yes	Homologous substitution
*PCDH15*	*ENSG00000150275.19*	*ENST00000320301.10*	1	−	−	C-terminal swap
*PCDH15*	*ENSG00000150275.19*	*ENST00000616114.4*	0.688	−	Yes	C-terminal swap
*PGM1*	*ENSG00000079739.17*	*ENST00000371083.4*	0.463	−	−	Homologous substitution
*PLEC*	*ENSG00000178209.16*	*ENST00000345136.8*	0.901	Yes	Yes	N-terminal swap
*RPGR*	*ENSG00000156313.15*	*ENST00000339363.7*	0.275	−	−	C-terminal swap
*SCN2A*	*ENSG00000136531.18*	*ENST00000636071.2*	0.922	−	Yes	Homologous substitution
*SCN8A*	*ENSG00000196876.18*	*ENST00000662684.1*	0.95	−	Yes	Homologous substitution
*STXBP1*	*ENSG00000136854.24*	*ENST00000373302.8*	0.955	−	Yes	Homologous substitution
*TP63*	*ENSG00000073282.14*	*ENST00000440651.6*	0.606	Yes	Yes	N-terminal swap
*TTN*	*ENSG00000155657.28*	*ENST00000360870.10*	0.302	−	Yes	Homologous substitution

The table lists those 32 alternative transcripts that contain at least one uniquely mapping ClinVar Pathogenic or Likely Pathogenic variant. Three genes (GNAS, PCDH15 and GCNT2) have two alternative transcripts with pathogenic variants. The list also shows the PubMed reference where possible, the TRIFID functional importance score [ref], and whether or not the transcripts is tagged as “MANE Plus Clinical” (MPC, [ref]). The expanded table is available in Supplementary Table 5.

### The clinical relevance of alternative isoforms

We have shown that APPRIS principal and MANE Select transcripts capture almost all pathogenic variants. However, some alternative protein isoforms are also biologically relevant^34,35^. How can researchers predict which alternative isoforms are clinically significant? Genes with validated pathogenic variants in alternative transcripts provide clues. The most obvious feature is that almost all alternative exons with pathogenic variants are ancient. For example, the alternative exon in *SLC25A3* pre-dates the earliest vertebrates^36^.

More than half of the 32 cases involve the alternative splicing of highly conserved tandem duplicated exons^36,37^, even though tandem duplicated exon substitutions make up <0.5% of annotated splice events^36^. Finally, alternative splicing is linked to tissue specificity at transcript^38^ and at protein level^31^. Tissue specificity also appears to be a characteristic of the alternative exons in this set^31^. Manual analysis found that only 20 of the 32 exon pairs (Table 1) had sufficient expression to determine tissue specificity^26^, but out of these 20 pairs of exons, 18 are clearly tissue specific. Two thirds of the set have both cross-species conservation and expression support.

### TRIFID functional importance scores predict clinical importance

A small number of clinically important alternative transcripts are labelled as MANE Plus Clinical^14^ based on ClinVar annotations. For example, the alternative transcript in *SLC25A3* is annotated as MANE Plus Clinical. Of the 58 MANE Plus Clinical transcripts from MANE v1.0, 23 map to the 32 pairs of Ensembl/GENCODE transcripts with pathogenic variants we validated (Supplementary Table 5), 21 correspond to genes in which the MANE Plus Clinical transcript has a uniquely mapped pathogenic variant, but the corresponding MANE Select transcript does not (as is the case of *SLC25A3*), and 12 transcripts either do not have uniquely mapped ClinVar pathogenic variants, or do not affect the alternative protein product (including *PTCH1*, for example).

We have demonstrated that reference transcripts produce the most clinically important protein isoform. Beyond this, the functional importance of alternative splice isoforms is clear only in a small number of genes^35^. We found certain features correlated with functional importance, so we developed TRIFID, a machine learning method that predicts the biological relevance of protein isoforms^39^. TRIFID inputs include conservation and expression data, and annotations from the Ensembl and APPRIS databases. We have shown that the TRIFID score can distinguish alternative exons that are under selective pressure from those that are not^39^. Here, we evaluated the ability of TRIFID to distinguish clinically important alternative transcripts.

We combined the 32 alternative exons from the set of genes with validated pathogenic variants in distinct transcripts with the 30 APPRIS alternative exons that have PubMed-supported pathogenic variants. Eleven exons appeared in both lists, so there were 51 alternative exons in all. To each of these exons we assigned the TRIFID score of the best-scoring transcript in which it was found. Just over half (27) had a TRIFID score of over 0.8, while only one had a TRIFID score below 0.2.

The distribution of TRIFID scores for all 76,134 APPRIS-defined alternative coding exons in GENCODE v37 was radically different. Again, we counted only the transcript with the best TRIFID score for each exon. Here, just 3.3% of alternative exons scored >0.8, and the overwhelming majority (almost 85%) had TRIFID scores below 0.2.

**Fig. 8:**
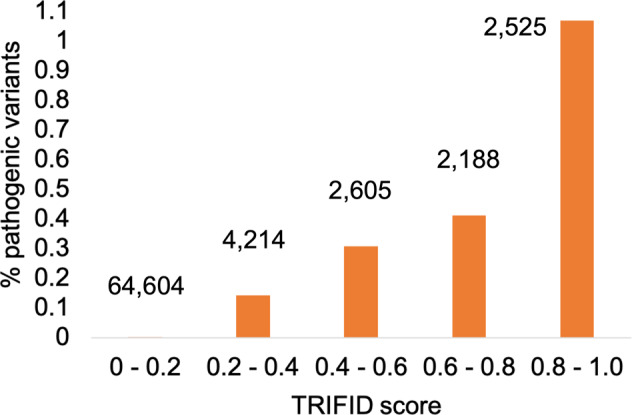
Validated pathogenic variants in alternative exons binned by TRIFID score. APPRIS alternative exons from the human reference set were binned by the TRIFID score of the best-scoring transcript in which they are annotated. For each set of exons in each bin, we show the percentage of exons that are annotated with validated pathogenic variants. The 51 validated pathogenic variants tend to fall in the highest scoring exons. Differences between the bins were huge, and significant despite the low numbers of validated pathogenic variants, Fisher exact tests showed that the percentage of pathogenic variants with best TRIFID scores exceeding 0.8 was significantly higher than those in all other bins (two tailed Fisher’s Exact test *p* = 0.0111 against the 0.6–0.8 bin, *p* = 0.001 against the 0.4–0.6 bin, and *p* < 0.00001 for the other two bins), and that the percentage of pathogenic variants among exons with TRIFID scores below 0.2 was significantly lower than all other bins (two tailed Fisher’s Exact test *p* < 0.00001 for all four bins).

We binned the TRIFID scores and calculated the proportion of the alternative exons with validated pathogenic variants in each bin (Fig. 8). We find that the higher the TRIFID score, the more likely an alternative transcript houses a validated pathogenic variant. In fact, exons from the highest scoring TRIFID alternative transcripts have 690 times as many validated pathogenic variants as the 85% of exons in the lowest scoring bin. More than 1% of alternative exons from transcripts with TRIFID scores >0.8 were annotated with validated pathogenic variants, but this fell to just 0.003% for exons from transcripts with TRIFID scores below 0.2.

### Limitations

The interpretation of pathogenic variants in Clinvar depends on the submitter, since submissions are not curated. There are a number of factors that might affect the quality of the interpretation, such as the date of submission and whether the variants are submitted by single submitters or large-scale prediction programs. In this analysis we included all pathogenic variants with a PubMed reference. This does not guarantee quality, but we manually curated pathogenic variants that mapped to alternative exons to create more reliable sets.

What we could not do was question the experimental process presented in each analysis. We did not reclassify the variants using ACMG criteria^1,2^. We did not attempt to reinterpret the analysis of the variants to determine whether they are truly pathogenic. This analysis should be carried out by the submitting group, or by clinical experts.

Manual curation of the papers allowed us to cross-check the evidence to see if it matched what was in ClinVar. For example, we could show that the coordinates in the paper that supported the pathogenic variant for *BNC2* at residue 853^32^ did not match the coordinates in ClinVar. For some variants we could check whether the assumptions made as part of the definition of pathogenicity were correct (as in the case of *NOBOX*). We could also check whether the pathogenic effect was reported for the annotated coding exon or for some other feature (as was the case with *HBD*). Finally, we could use evidence that was not considered by the authors to suggest that the pathogenic label is erroneous (as with the variant in *ACTG2*).

The ACMG criteria were first published in 2008^1^, so one possibility is that authors were laxer with their submissions prior to 2008. We found no evidence of this in our (limited) set. Just two possible pathogenic variant misannotations pre-dated these recommendations (*KCNQ2* and *LDB3*), and one of these was a simple mis-annotation of protein coordinates^18^.

## Discussion

In depth analysis of pathogenic variants from the ClinVar database shows that almost all valid pathogenic variants can be mapped to one reference transcript per gene. Very few ClinVar pathogenic variants map to alternative exons. In addition, we find that this reference transcript almost always corresponds to the APPRIS principal isoform^15^ and the MANE Select transcript^14^. Over genes in which APPRIS and MANE agreed on the reference transcript, just 13 validated pathogenic variants (0.042%) mapped to alternative exons.

The biological importance of MANE Select and APPRIS principal transcripts is underlined by data from human genetic variation and large-scale proteomics experiments. APPRIS principal isoforms and the isoforms produced by MANE Select transcripts agree with the main proteomics isoform in >98% of genes^40^. In addition, MANE Select and APPRIS principal exons are under purifying selection, while exons unique to the longest CDS are not^40^^,41^.

The American College of Medical Genetics and Genomics (ACMG) recommends the longest or most clinically significant RefSeq transcript as the reference transcript^2^. However, using the longest transcript has no biological justification. We find that choosing the transcript with the longest coding sequence as the reference is a suboptimal solution because it captures significantly fewer validated pathogenic variants than the MANE Select or APPRIS principal transcripts.

Instead, we recommend that APPRIS principal and MANE Select transcripts be used as the reference transcripts in clinical variant interpretation. In GENCODE v37, APPRIS principal and MANE Select transcripts agreed over 94% of genes. Genes in which APPRIS and MANE select the same reference transcript will be listed on the APPRIS website.

Over the few genes where they disagree (for example *TAFAZZIN, NOBOX* and *LDB3*), researchers may have to use further criteria to choose a reference transcript. TRIFID^39^, for example, can identify likely functional alternative isoforms. Since both APPRIS and MANE have been developed in conjunction with the GENCODE Consortium, disagreements should become fewer with time.

For alternative transcripts, we find that many known pathogenic variants are annotated as MANE Plus Clinical transcripts. We show that TRIFID functional importance scores can identify clinically relevant alternative transcripts. Alternative exons from the highest scoring transcripts are almost 700 times more likely to harbour pathogenic variants than these low scoring transcripts.

We find that pathogenic variants that map to alternative exons can be erroneously annotated (*BNC2*, *KCNQ2*, *PTCH1*) or may affect non-coding regions on top of which alternative coding exons have been annotated (*HBD*, *SDCCAG8*, *FECH*, *CYP21A2*). The first set of variants can be corrected via curation, but the second set of pathogenic variants are correct. Here it is the predicted alternative coding exon that should be removed. Coding exons ought not to be annotated on top of important non-coding features.

We have demonstrated that MANE Select and APPRIS principal transcripts best predict the most clinically relevant transcript, particularly when they agree. Clinical and biomedical researchers ought to use these two methods rather than the longest transcript when selecting reference transcripts.

## Methods

### MANE select transcripts

MANE Select and MANE Plus Clinical transcripts^14^ are annotated in the gtf file of the GENCODE v37 reference human gene set^17^, which can be found at https://www.gencodegenes.org/human/. MANE Select transcripts are transcripts that are annotated both in the RefSeq and in the Ensembl human gene set. They have to coincide exactly in the coordinates of both the coding exons (CDS) and the 3′ and 5′ untranslatable regions (UTR) regions. Where there is more than one transcript that fits this definition, the MANE Select transcript is chosen from two separate prediction pipelines, one in RefSeq and one in Ensembl. The two pipelines are similar in that they use information such as the UniProtKB display isoform, expression data, ClinVar variants, LCG definition, though they do have differences. For example, the APPRIS principal isoform is an input to the Ensembl prediction pipeline, but not the RefSeq prediction pipeline. More details of the pipeline are available in the paper. Where no single agreed transcript exists, RefSeq and Ensembl manual annotation teams work together to choose one transcript as the MANE Select transcript. The MANE Select version we used in this analysis was v0.95. This was the version in GENCODE v37. MANE Select has now been updated to v1.0. We mapped the ClinVar variants to just the CDS of the MANE transcripts.

### The longest transcripts

The ACMG recommends the use of the longest transcript as the reference transcript. It is not 100% clear from their recommendations how the longest transcript should be chosen. We selected the longest transcript in two different ways, and used the more efficient of the two methods in our analysis. First, we selected the longest protein coding transcript using both UTR and CDS, and second we selected the longest protein coding transcript using just CDS. While the two transcripts were the same in about 70% of the genes, they differed in the remaining genes. The transcript with the longest CDS missed considerably fewer pathogenic variants before curation than the transcript with the most CDS and UTR bases (160 versus 1,967, a factor of 12). So, in the end, we mapped the ClinVar variants to the exons from the transcripts with the longest CDS in each gene in the GENCODE v37 gene set.

### APPRIS principal transcripts

APPRIS principal isoforms are selected based on cross-species conservation and the preservation of structural and functional features. APPRIS has four main modules that map protein structure, functional domains, ligand-binding residues and cross-species conservation to isoforms. Where the data from the four modules are not able to select a clear principal isoform, the decision is made based on external methods such as TRIFID^39^ and proteomics data^31^.

The APPRIS principal isoforms^15^ for the GENCODE v37 gene set^17^ for each annotated isoform were downloaded from the APPRIS database (https://appris.bioinfo.cnio.es/). The principal isoforms are selected at the protein level, but each is referenced to an Ensembl/GENCODE transcript, and it is this transcript that we use to represent the “principal transcript” in this analysis. Because genes are often annotated with transcripts that have identical CDS, but distinct UTR, there can be more than one (coding sequence identical) principal transcript/isoform per gene.

### TRIFID functional isoform prediction

TRIFID is a machine learning algorithm that predicts the likely functional importance of protein isoforms. It was trained on protein isoforms detected in large-scale tissue-based proteomics analysis, and bases its predictions on features from the APPRIS database, the Ensembl/GENCODE annotation, the Pfam database and data from a large-scale RNAseq experiment^42^. Features based on cross-species conservation are the most powerful predictors in TRIFID. The TRIFID score (between 0 and 1) represents the likely functional relevance of protein isoforms and testing shows that exons from high scoring transcripts are under selective pressure, while alternative exons from low scoring isoforms are generally not^39^. TRIFID functional importance scores for the predicted GENCODE v37 isoforms were calculated as part of the APPRIS principal isoform selection pipeline.

### Analysis of pathogenic variants

ClinVar variants were extracted from raw VCF files that were downloaded on 4 April 2021. The use of this publicly available large-scale data does not require ethical approval. We mapped the variants to the coding genes and transcripts annotated in v37 of the GENCODE human reference set using VEP^43^. A total of 656,237 ClinVar variants mapped to GENCODE coding transcripts. Along with each variant, we recorded the official ClinVar CLIN_SIG designation, and whether or not the variant was supported by a reference in PubMed, or was reviewed by an expert panel.

Many variants mapped to more than one transcript. In these cases, we reduced the mapping to a single transcript. For the APPRIS analysis, we retained the mapping to the APPRIS principal transcript where possible. For the MANE analysis, we prioritised the mapping to the MANE Select transcript, and for the longest CDS analysis we prioritised the longest CDS. Variants that did not map to a principal, MANE Select or longest transcript were associated to one of the alternative transcripts that they mapped to—in each case the alternative transcript with the highest TRIFID score.

For the various analyses of pathogenic variants, we defined pathogenic variants as those tagged as Pathogenic, Likely Pathogenic or Pathogenic/Likely Pathogenic by ClinVar. For the initial analysis, we also required that each of these pathogenic variants also had a supporting PubMed publication. This was to allow us to cross-check the evidence for pathogenicity for each variant. The Richards et al. ACMG analysis^2^ was excluded as a supporting PubMed publication; although this paper is used as a reference for 25,382 variants, the paper does not provide verifiable information about any of these variants.

### Manual curation

Much of the analysis in this paper was manual. The correspondence between pathogenic variants in research papers and ClinVar annotations was checked via the individual papers. We checked whether the information in the paper was correct, and confirmed that the coordinates matched those of ClinVar. Gene model structures were analysed using the Ensembl web browsers^13^, and the APPRIS database^15^. Conservation of exons was calculated from BLAST searches against genomes in RefSeq^3^ and UniProtKB^7^, and splice site conservation was analysed with CodeAlignView (https://www.data.broadinstitute.org/compbio1/cav.php?). Expression evidence from the Gtex consortium^43^^,44^ was analysed using the GNOMAD^26^ web pages. Protein sequences and variants were mapped onto 3D structures from the AlphaFold-EBI collaboration^45^, and the PDB^46^.

### Reporting summary

Further information on research design is available in the Nature Research Reporting Summary linked to this article.

## Supplementary information

Supplementary Information

Supplementary Tables

Reporting Summary

## Data Availability

MANE Select and Plus Clinical transcripts annotations can be downloaded with the Ensembl (https://www.ensembl.org/index.html), GENCODE (https://www.gencodegenes.org/human/) and RefSeq (https://www.ncbi.nlm.nih.gov/refseq/) annotations of the human and mouse gene sets. APPRIS principal isoforms and TRIFID functional importance scores for human gene sets, along with a range of other vertebrate and invertebrate model species are available from the APPRIS website (https://appris.bioinfo.cnio.es/). They are available for Ensembl and RefSeq gene sets, and for both GRCh37 and GRCh38 builds. APPRIS principal isoforms/transcripts can also be downloaded from Ensembl/GENCODE. ClinVar variants can be downloaded from the NIH web site (https://www.ncbi.nlm.nih.gov/clinvar/).

## Code availability

All local scripts to map the variants to the longest CDS, APPRIS principal and MANE Select exons were written in Perl, and are available on request from the authors. VEP (https://www.ensembl.org/info/docs/tools/vep/index.html) was used to map ClinVar variants to the GENCODE v37 gene set.
