# Supplementary material for: Clinical variant interpretation and biologically relevant reference transcripts
Source: NPJ Genom Med. 2022 Oct 18;7:59. doi: 10.1038/s41525-022-00329-6 (PMC9579139; doi:10.1038/s41525-022-00329-6)
Supplement: Supplementary file 1 — Supplementary Information [file 41525_2022_329_MOESM1_ESM.docx]

**Supplementary Table 1. Pathogenic variants with PubMed support that map to exons unique to APPRIS alternative transcripts**

The notes column shows whether the variant had an effect on the alternative protein isoform, and if we found that it did not, why.

**Supplementary Table 2. Pathogenic variants with PubMed support that map to exons unique to MANE Select alternative transcripts**

The notes column shows whether the variant had an effect on the alternative protein isoform, and if we found that it did not, why.

**Supplementary Table 3. Pathogenic variants with PubMed support that map to exons unique to transcripts alternative to the longest CDS**

The result column shows whether the variant had an effect on the alternative protein isoform, and if we found that it did not, why.

**Supplementary Table 4. Pathogenic variants with PubMed support that map to exons unique to transcripts alternative to APPRIS principal and MANE Select transcripts, over those genes where the two methods agree**

The notes column shows whether the variant had an effect on the alternative protein isoform, and if we found that it did not, why.

**Supplementary Table 5. ClinVar pathogenic variants that map to exons unique to alternative transcripts that we validated**

The main transcript here was determined by mapping as many pathogenic variants to one transcript as possible. Best TRIFID score is the functional importance score for the alternative transcript, as described in the paper. Protein effect shows the change undergone in the alternative protein. MXE = mutually exclusively spliced tandem duplicated (homologous) exon.

**Supplementary Table 6. Pathogenic variants that map to exons unique to alternative transcripts and that we did not validate**

The main transcript here was determined by mapping as many pathogenic variants to one transcript as possible. Primate transposon indicates which transcripts overlap SINE Alu or LINE regions. Protein effect shows the change undergone in the alternative protein.
